# Comparison of the Respiratory Resistomes and Microbiota in Children Receiving Short versus Standard Course Treatment for Community-Acquired Pneumonia

**DOI:** 10.1128/mbio.00195-22

**Published:** 2022-03-24

**Authors:** M. M. Pettigrew, J. Kwon, J. F. Gent, Y. Kong, M. Wade, D. J. Williams, C. B. Creech, S. Evans, Q. Pan, E. B. Walter, J. M. Martin, J. S. Gerber, J. G. Newland, M. E. Hofto, M. A. Staat, V. G. Fowler, H. F. Chambers, W. C. Huskins

**Affiliations:** a Department of Epidemiology of Microbial Diseases, Yale School of Public Health, New Haven, Connecticut, USA; b Department of Environmental Health Sciences, Yale School of Public Health, New Haven, Connecticut, USA; c Department of Biostatistics, Yale School of Public Health, New Haven, Connecticut, USA; d Department of Molecular Biophysics and Biochemistry, W.M. Keck Foundation Biotechnology Resource Laboratory, Yale School of Medicine, New Haven, Connecticut, USA; e Department of Pediatrics and the Vanderbilt Vaccine Research Program, Vanderbilt University School of Medicine, Monroe Carell Jr. Children’s Hospital at Vanderbilt, Nashville, Tennessee, USA; f Biostatistics Center, Milken Institute School of Public Health, George Washington University, Washington, DC, USA; g Department of Pediatrics, Duke Human Vaccine Institute, Duke University School of Medicine, Durham, North Carolina, USA; h Department of Pediatrics, University of Pittsburgh School of Medicine, the UPMC Children’s Hospital of Pittsburgh, Pittsburgh, Pennsylvania, USA; i Children’s Hospital of Philadelphia, Philadelphia, Pennsylvania, USA; j Department of Pediatrics, Perelman School of Medicine at the University of Pennsylvania, Philadelphia, Pennsylvania, USA; k Department of Pediatrics, Washington University in St. Louis School of Medicine, St. Louis, Missouri, USA; l Department of Pediatrics, University of Alabama at Birmingham School of Medicine, Birmingham, Alabama, USA; m Cincinnati Children’s Hospital Medical Center, Department of Pediatrics, University of Cincinnati College of Medicine, Cincinnati, Ohio, USA; n Department of Medicine and Duke Clinical Research Institute, Duke University School of Medicine, Durham, North Carolina, USA; o Department of Medicine, University of California San Francisco, San Francisco, California, USA; p Mayo Clinic College of Medicine and Science and the Department of Pediatric and Adolescent Medicine, Mayo Clinic, Rochester, Minnesota, USA; Rutgers University

**Keywords:** microbiota, resistome, antibiotic resistance, community-acquired pneumonia, children, respiratory tract infections

## Abstract

Pediatric community-acquired pneumonia (CAP) is often treated with 10 days of antibiotics. Shorter treatment strategies may be effective and lead to less resistance. The impact of duration of treatment on the respiratory microbiome is unknown. Data are from children (*n* = 171), ages 6 to 71 months, enrolled in the SCOUT-CAP trial (NCT02891915). Children with CAP were randomized to a short (5 days) versus standard (10 days) beta-lactam treatment strategy. Throat swabs were collected at enrollment and the end of the study and used for shotgun metagenomic sequencing. The number of beta-lactam and multidrug efflux resistance genes per prokaryotic cell (RGPC) was significantly lower in children receiving the short compared to standard treatment strategy at the end of the study (Wilcoxon rank sum test, *P* < 0.05 for each). Wilcoxon effect sizes were small for beta-lactam (*r*: 0.15; 95% confidence interval [CI], 0.01 to 0.29) and medium for multidrug efflux RGPC (*r*: 0.23; 95% CI, 0.09 to 0.37). Analyses comparing the resistome at the beginning and end of the trial indicated that in contrast to the standard strategy group, the resistome significantly differed in children receiving the short course strategy. Relative abundances of commensals such as Neisseria subflava were higher in children receiving the standard strategy, and *Prevotella* species and Veillonella parvula were higher in children receiving the short course strategy. We conclude that children receiving 5 days of beta-lactam therapy for CAP had a significantly lower abundance of antibiotic resistance determinants than those receiving standard 10-day treatment. These data provide an additional rationale for reductions in antibiotic use when feasible.

## INTRODUCTION

Community-acquired pneumonia (CAP) is a common childhood illness and is estimated to result in approximately 1 million antibiotic prescriptions annually in the United States (1, 2). Antibiotic treatment may be associated with adverse effects that include dysbiosis in the microbiome (3–5). Pediatric CAP is generally treated with 10 days of antibiotics in North America (6, 7). Treatment strategies involving shorter courses of antibiotics have been proposed to reduce side effects and decrease antibiotic selection pressure on the microbiome (5, 8). Recent clinical trials have suggested that 5 days are as effective as 10 days of therapy for pediatric CAP (6, 7). However, limited data have examined explicitly the relationship between the duration of antibiotic therapy and its impact on the respiratory resistome (i.e., the community of antibiotic resistance genes) (9).

SCOUT-CAP is a multicenter, randomized, double-blind, placebo-controlled, superiority clinical trial that compared a 5-day (short) beta-lactam strategy to a 10-day (standard) beta-lactam strategy for the treatment of pediatric CAP (trial registration number: NCT02891915) (7). The current substudy evaluated changes in the microbiome (antibiotic resistome and microbiota) in SCOUT-CAP participants who received short and standard durations of antibiotic therapy. We used shotgun metagenomic sequencing to compare the resistomes and microbiota in throat samples from children receiving short versus standard treatment strategies at the enrollment visit and at the end of the study (i.e., 19 to 25 days after the initiation of the study drug). The resistomes of the gastrointestinal tract were also compared between treatment strategy groups. Our primary hypothesis was that the abundance of respiratory beta-lactam antibiotic resistance genes (ARGs) would be lower at the end of the study in children receiving short-duration therapy compared to those who received standard durations of antibiotics.

## RESULTS

### Abundance and diversity of respiratory ARGs at the end of the study.

**FIG 1:**
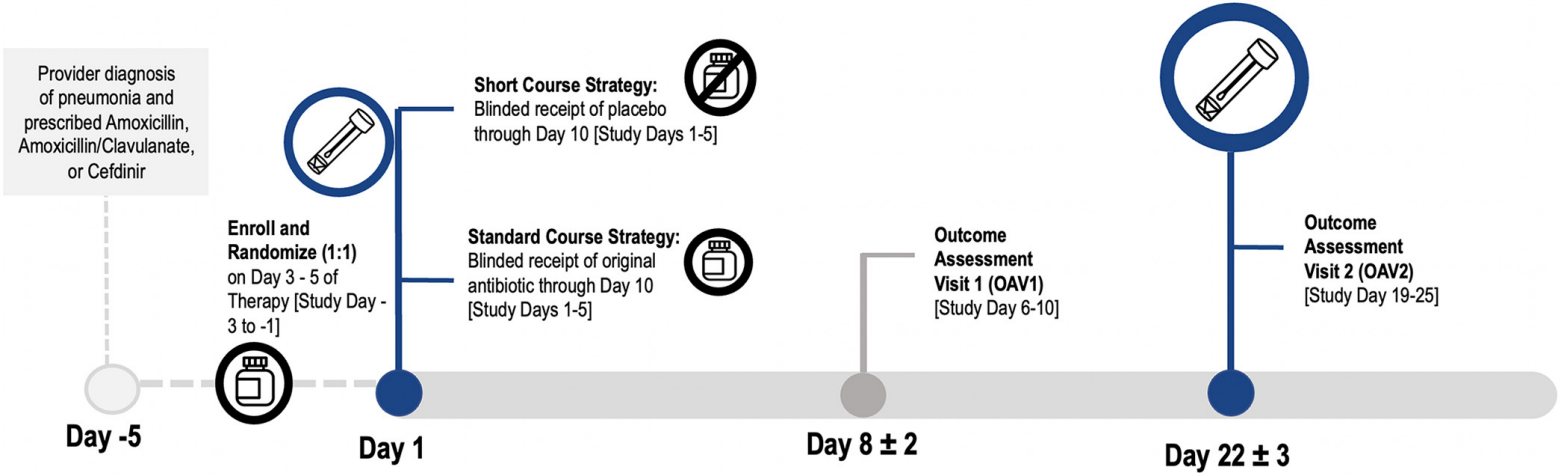
SCOUT-CAP study design and timeline. SCOUT-CAP was a multicenter, randomized, double-blind, placebo-controlled, superiority clinical trial, which evaluated a short course (5 days) versus standard course (10 days) strategy of beta-lactam therapy for outpatient treatment of pediatric CAP. Participants were enrolled on days 3 to 6 of their initially prescribed oral beta-lactam therapy and randomized to 5 days of matching placebo (short course strategy) or an additional 5 days of their prestudy antibiotic (standard course strategy). Outcome assessment visits (OAV) occurred on study day 6 to 10 (OAV1) and study day 19 to 25 (OAV2). Throat swabs used in this study were collected at enrollment and OAV2.

The overall study design, timelines for sample collection, and antibiotic treatment strategies are shown in Fig. 1. Our initial analyses focused on the intention-to-treat (ITT) population; we compared the abundance of respiratory ARG types in children randomized to short versus standard treatment strategies at the end of the study (i.e., outcome assessment visit [OAV2]). Throat samples were obtained and evaluated for ARGs for 171 subjects. Baseline characteristics are summarized in Table 1 and were similar to those reported for the 380 children in the ITT population of the SCOUT-CAP trial (7). The average age was 35 months (standard deviation [SD], 17.6). Ninety-one percent of participants were prescribed amoxicillin as their initial antibiotic followed by amoxicillin-clavulanate (6%) and cefdinir (2%). The short course and standard strategy groups did not significantly differ by age, sex, race, ethnicity, or initial antibiotic prescribed.

**TABLE 1:** Baseline characteristics of the study population^*a*^

Characteristic	Short course (*n* = 84), no. (%)	Standard course (*n* = 87), no. (%)	Total (*n* = 171), no. (%)
Age			
6–23 mo	26 (31)	30 (34)	56 (33)
24–71 mo	58 (69)	57 (66)	115 (67)
Sex			
Female	46 (55)	38 (44)	84 (49)
Male	38 (45)	49 (56)	87 (51)
Race			
Asian	4 (5)	1 (1)	5 (3)
Black or African American	19 (23)	23 (26)	42 (25)
Multiracial	8 (10)	4 (5)	12 (7)
White	51 (61)	58 (67)	109 (64)
Unknown	2 (1)	1 (1)	3 (2)
Ethnicity			
Hispanic or Latino	8 (10)	10 (12)	18 (10)
Not Hispanic or Latino	75 (89)	77 (88)	152 (89)
Unknown	1 (1)	0 (0)	1 (1)
Initial antibiotic			
Amoxicillin	78 (93)	78 (90)	156 (91)
Amoxicillin-clavulanate	4 (5)	7 (8)	11 (6)
Cefdinir	2 (2)	2 (2)	4 (2)

a Data are presented as no. (%) unless otherwise specified. There were no statistically significant differences observed for any of the variables tested when comparing frequencies between the standard and the short treatment strategies (*P* > 0.05 for each).

**FIG 2:**
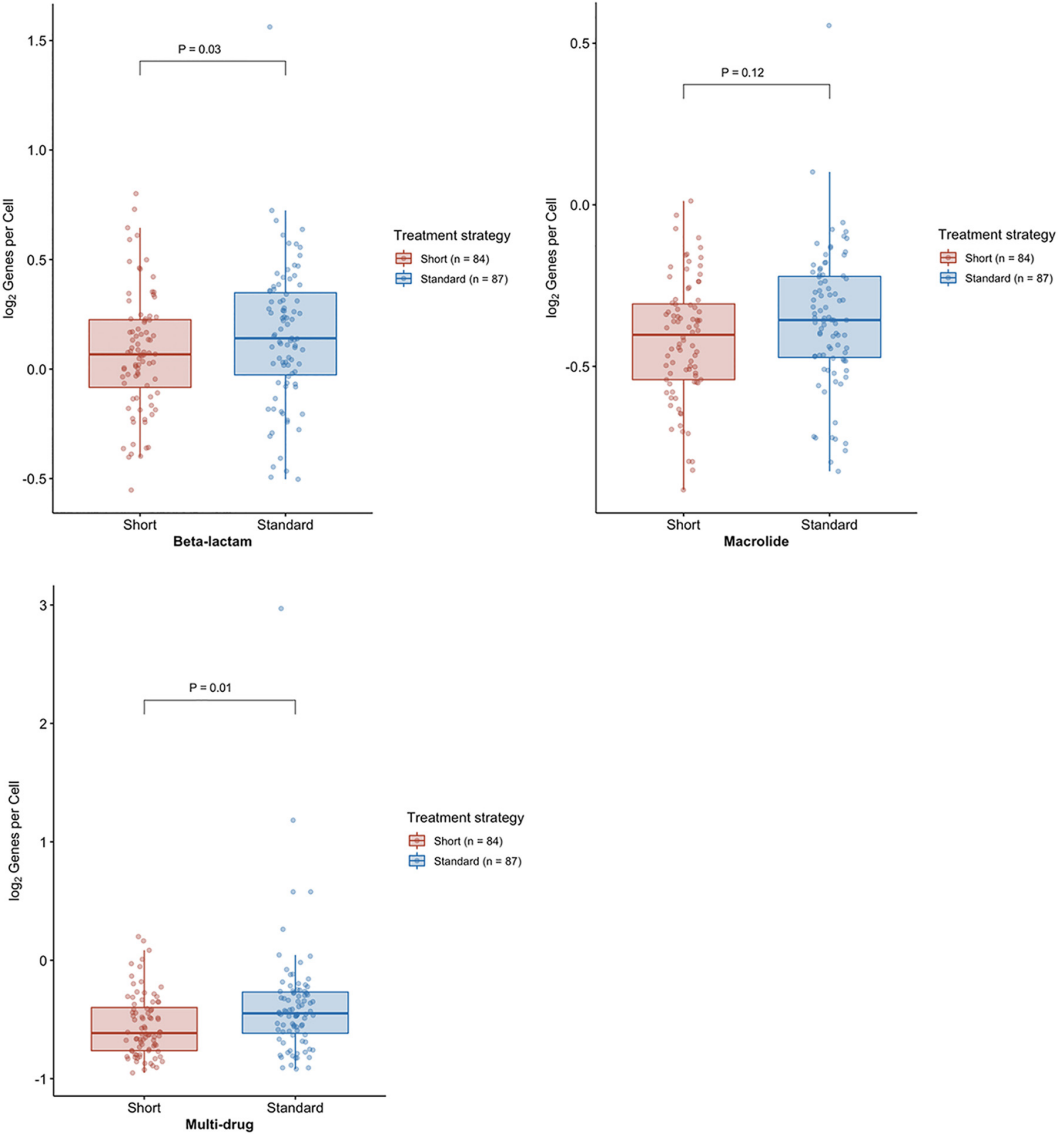
Boxplot of beta-lactam, macrolide, and multidrug efflux resistance genes per prokaryotic cell (RGPC) in throat swabs from 171 participants at the end of the study. The boxplots depict the distribution of RGPC for beta-lactam (top left), macrolide (top right), and multidrug efflux (bottom left) resistance genes. The line reflects the median RGPC, lower and upper hinges correspond to the first and third quartiles, respectively, and upper and lower whiskers extend from the hinge to the highest value that is within 1.5× interquartile range of the hinge. A one-sided Wilcoxon rank sum test was used to assess statistically significant differences (alpha level <0.05) between the numbers of RGPC in participants assigned to a short course (red) or standard course (blue) strategy. Log_2_ normalized RGPC data were used for visualization.

As shown in Fig. 2, the median abundance of respiratory beta-lactam resistance genes per prokaryotic cell (RGPC) was significantly lower at OAV2 in the short course group than in the standard course strategy group. The median abundance of beta-lactam RGPC was 0.55 (range 0.18 to 1.24) and 0.60 (range 0.21 to 2.45) for short course and standard course strategy groups, respectively (7). The Wilcoxon effect sizes of these differences were small for beta-lactam RGPC (*r*: 0.15; 95% confidence interval [CI], 0.01 to 0.29). We compared the median RGPC for nine additional clinically relevant ARGs by treatment strategy groups; the median RGPC of macrolide and multidrug efflux ARGs were lower in the short course strategy group than in the standard strategy group. The Wilcoxon effect size was small for macrolide RGPC (*r*: 0.15; 95% CI, 0.02 to 0.29) and medium for multidrug efflux ARGs (*r*: 0.23; 95% CI, 0.09 to 0.37). After using the Benjamini-Hochberg procedure to derive false-discovery rate (FDR)-adjusted *P* values, the differences in multidrug efflux RGPC remained statistically significant (FDR-adjusted *P* = 0.01). However, differences in macrolide RGPC were no longer significantly different by treatment group after FDR adjustment (FDR-adjusted *P* = 0.12) (Fig. 2). The median abundance of multidrug efflux RGPC was 0.15 (range 0.02 to 0.65) and 0.23 (range 0.03 to 7.34) for short course and standard course strategy groups, respectively. There were no significant differences identified for the other seven types of resistance genes examined.

ARG subtype analyses resulted in the identification of 387 individual resistance genes within our OAV2 samples; there were no significant differences in subtype-level RGPC by treatment groups. Examples of transmissible resistance genes included TEM-1 and ROB-1 beta-lactamases, which have been shown to contribute to resistance in Haemophilus influenzae (10), and *mec*A, which encodes beta-lactam resistance in Staphylococcus species. A list of beta-lactam, macrolide, and multidrug efflux ARGs at the subtype level is provided in Data Set S1 in the supplemental material.

10.1128/mbio.00195-22.4 DATA SET S1 Data set providing the resistance genes per prokaryotic cell (RGPC) at the individual gene (i.e., subtype) level for beta-lactam, macrolide, and multidrug efflux antibiotic resistance genes. Data are listed by a deidentified specimen number and treatment strategy group. Download Data Set S1, XLSX file, 0.3 MB. Copyright © 2022 Pettigrew et al. 2022 Pettigrew et al. https://creativecommons.org/licenses/by/4.0/ This content is distributed under the terms of the Creative Commons Attribution 4.0 International license.

Next, we examined the diversity of ARGs at OAV2 for 171 subjects in the ITT population. Used in our context, the Shannon diversity index provides a measure of the number of different types of ARGs within each sample while taking their abundance and evenness (distribution of abundance) into account (11). Median Shannon diversity was 1.37 (range 1.10 to 1.84) and 1.42 (0.85 to 1.78) for the short course and standard course strategies, respectively. Median Shannon diversity did not differ by treatment strategy (two-sided Wilcoxon rank sum test, *P* = 0.92). The Simpson diversity index describes the probability that two randomly selected sequence reads map to the same resistance gene, and abundant ARGs are given more weight (11). Median inverse Simpson values were 3.12 (range 2.30 to 5.06) and 3.22 (range 1.55 to 5.38) for the short course and standard course strategies, respectively. Median inverse Simpson indices did not differ by treatment strategy (two-sided Wilcoxon rank sum, *P* = 0.48).

As per the SCOUT-CAP study protocol, all children were categorized as having had at least 5 days of beta-lactam therapy (7). Children receiving the short course strategy had a median of 5 days on antibiotics (range 5 to 12 days) prior to OAV2. Children in the standard treatment group had a median of 10 days of antibiotic therapy (range 9 to 16 days). Four children, one in the short course strategy group and three in the standard course strategy group, received additional antibiotics for other indications between the enrollment visit and OAV2. We conducted an according-to-protocol analysis (ATP) of 152 children, which excluded the four children who received additional antibiotics for other indications and an additional 15 children who either did not take the study drug as directed and/or had throat samples taken more than ±48 h outside the scheduled OAV2 study visit. Beta-lactam and multidrug efflux RGPC were lower in children receiving the short compared to standard treatment strategy (Wilcoxon rank sum test, FDR-adjusted *P* = 0.05 and 0.03, respectively).

### Temporal changes in the respiratory resistome.

**FIG 3:**
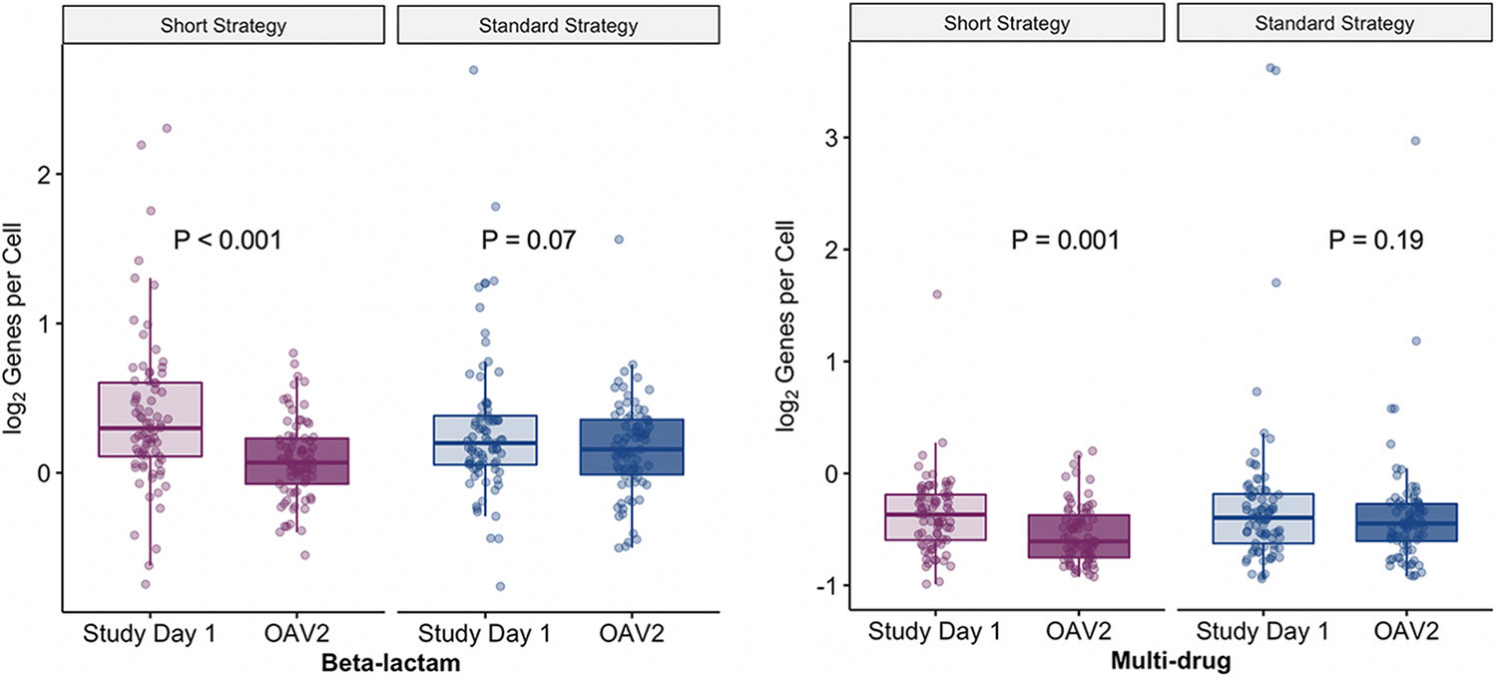
Comparison of abundances of beta-lactam and multidrug efflux resistance genes per prokaryotic cell (RGPC) at the enrollment visit and end of the study by treatment strategy (*n* = 158). Boxplots of beta-lactam (left) and multidrug efflux (right) resistance genes per prokaryotic cell (RGPC) in throat swabs from 158 participants at the enrollment visit and end of the study stratified by treatment strategy. The line reflects the median RGPC, lower and upper hinges correspond to the first and third quartiles, respectively, and upper and lower whiskers extend from the hinge to the highest value that is within 1.5× interquartile range of the hinge. A one-sided Wilcoxon signed-rank test was used to assess statistically significant differences (alpha level <0.05) between the numbers of RGPC in participants at enrollment (study day 1) and at the end of the study (outcome assessment visit 2 [OAV2]); participants were stratified by assignment to a short course (light and dark purple) or standard course (light and dark blue) strategy. FDR-adjusted *P* values are shown. Log_2_ normalized RGPC data were used for visualization.

We also sought to describe the resistome over time. Paired enrollment and OAV2 throat samples were available for 158 participants. As expected at baseline (i.e., the enrollment visit), there were no significant differences by treatment strategy in the abundance of RGPC for any of the 10 antibiotic types examined (Fig. S1). Since the abundances of beta-lactam and multidrug efflux RGPC were shown to differ at OAV2, we compared the RGPC for these two ARG types at the enrollment visit and OAV2 stratified by treatment strategy (Fig. 3). The abundances of beta-lactam and multidrug efflux RGPC were significantly lower at OAV2 than at the enrollment visit for children receiving the short course strategy. In contrast, there were no significant differences in the RGPC for these ARG types in children receiving the standard course strategy.

10.1128/mbio.00195-22.1 FIG S1 Boxplot of respiratory resistance genes per prokaryotic cell (RGPC) for 10 clinically relevant ARG types in throat swabs at enrollment (*n* = 158). Comparison of the abundances of resistance genes per prokaryotic cell (RGPC) for 10 clinically relevant antibiotic resistance gene (ARG) types in throat swabs at enrollment (*n* = 158). A two-sided Wilcoxon rank sum test was used to assess statistically significant differences (alpha level <0.05) between the numbers of RGPC in participants assigned to a short course (purple) or standard course (blue) strategy. FDR-adjusted *P* values are shown. Download FIG S1, DOCX file, 1.2 MB. Copyright © 2022 Pettigrew et al. 2022 Pettigrew et al. https://creativecommons.org/licenses/by/4.0/ This content is distributed under the terms of the Creative Commons Attribution 4.0 International license.

**FIG 4:**
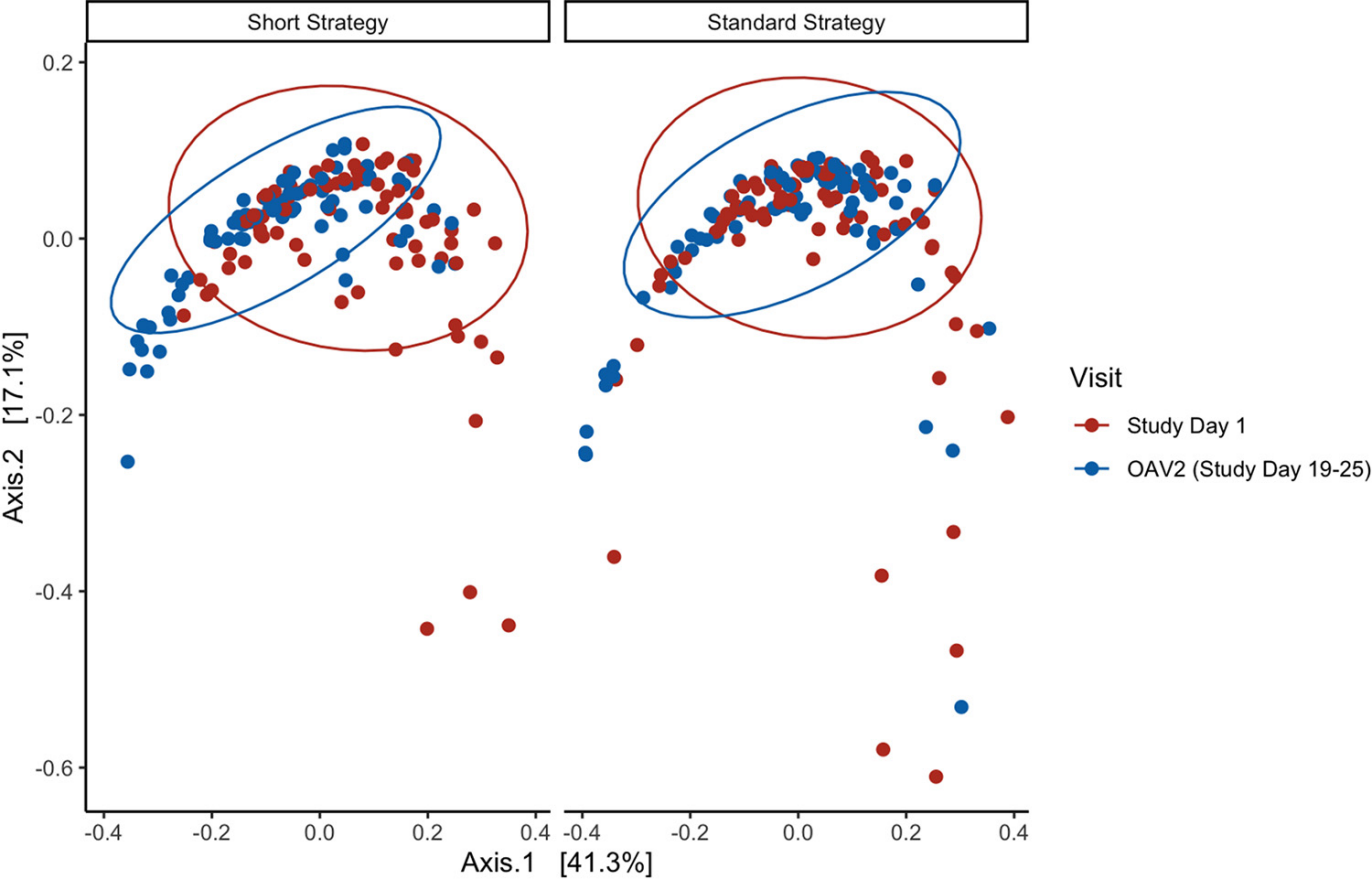
Principal-coordinate analysis (PCoA) plot of Bray-Curtis dissimilarity of the resistome at enrollment and OAV2 by treatment strategy (*n* = 158). Principal-coordinate analysis (PCoA) plot of Bray-Curtis dissimilarity of all type-level resistance genes per prokaryotic cell (RGPC) (i.e., the resistome) at enrollment (study day 1) and end of the study (outcome assessment visit 2 [OAV2]) stratified by treatment strategy. Study day 1 is shown in red, and OAV2 is shown in blue.

Next, we were interested in examining global compositional differences in the resistome over time and by treatment group. Bray-Curtis dissimilarity indices were calculated, and principal-coordinate analysis (PCoA) was used to visualize the differences in the community of ARGs (i.e., the resistome) at enrollment and OAV2 by treatment strategy (Fig. 4). Permutational multivariate analysis of variance analyses (PERMANOVA) indicated that there was a statistically significant difference in the compositional profile of the resistome when comparing the enrollment visit and OAV2 (*P* < 0.001). The compositional profile of the resistome did not significantly differ when comparing the short course and standard course groups; however, we identified a significant interaction between treatment strategy and visit (*P* = 0.03), which suggested that the level of compositional dissimilarity at enrollment and OAV2 differed by treatment strategy. Thus, we stratified by treatment strategy. The stratified analysis showed that there was a significant difference in the compositional profile of the resistome at enrollment and OAV2 in children receiving the short course strategy (FDR-adjusted *P* < 0.001). The observed differences were not due to heterogeneity in variance as measured by multivariate homogeneity of group dispersions (*P* > 0.05). In contrast, compositional dissimilarity in the resistome did not significantly differ between enrollment visit and OAV2 in children receiving the standard course strategy (FDR-adjusted *P* = 0.09).

### Taxonomic characterization of the respiratory microbiota at the end of the study.

**FIG 5:**
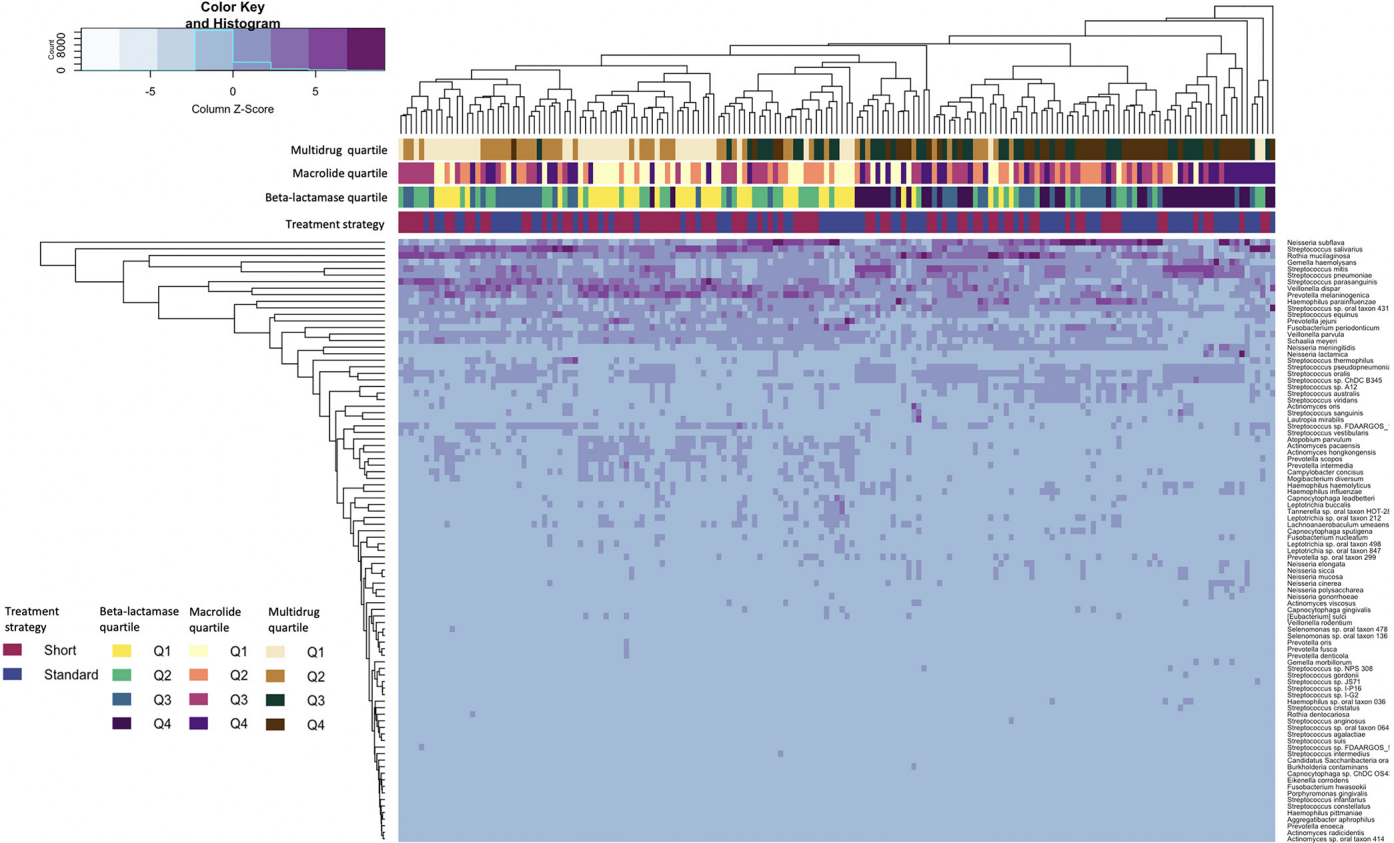
Heatmap comparing the abundances of 92 bacterial species in 171 children at outcome assessment visit 2 (OAV2). Column-scaled heatmap of log_2_ transformed relative abundance of the 92 prevalence-filtered bacterial taxa identified in throat samples of 171 children at OAV2 (color key is indicated in the upper left corner). Complete linkage clustering of samples was based on the species and abundance. Bars represent treatment strategy assignment and quartiles of beta-lactamase, macrolide, and multidrug resistance gene types; the color key is indicated in the lower left corner.

Taxonomic classification of the shotgun metagenomic sequence data resulted in the identification of 4,494 unique species. Ninety-two unique species remained after prevalence filtering (Fig. 5). We used two different analytic methods to identify differentially abundant taxa in the short course and standard course treatment strategy groups. Linear discriminant analysis effect size (LEfSe) (12) analyses showed that the relative abundance of Neisseria subflava, *Capnocytophaga* species ChDC OS43, and Neisseria cinerea were higher in the standard course strategy group. Relative abundances of Prevotella scopos, Prevotella oris, Prevotella jejuni, and Veillonella parvula were higher in the short course strategy group. Analysis of compositions of microbiome with bias correction (ANCOM-BC) (13) identified *N. subflava* as more abundant in the standard course strategy group; after FDR adjustment, there were no taxa identified by ANCOM-BC that differed in abundance by treatment strategy.

**FIG 6:**
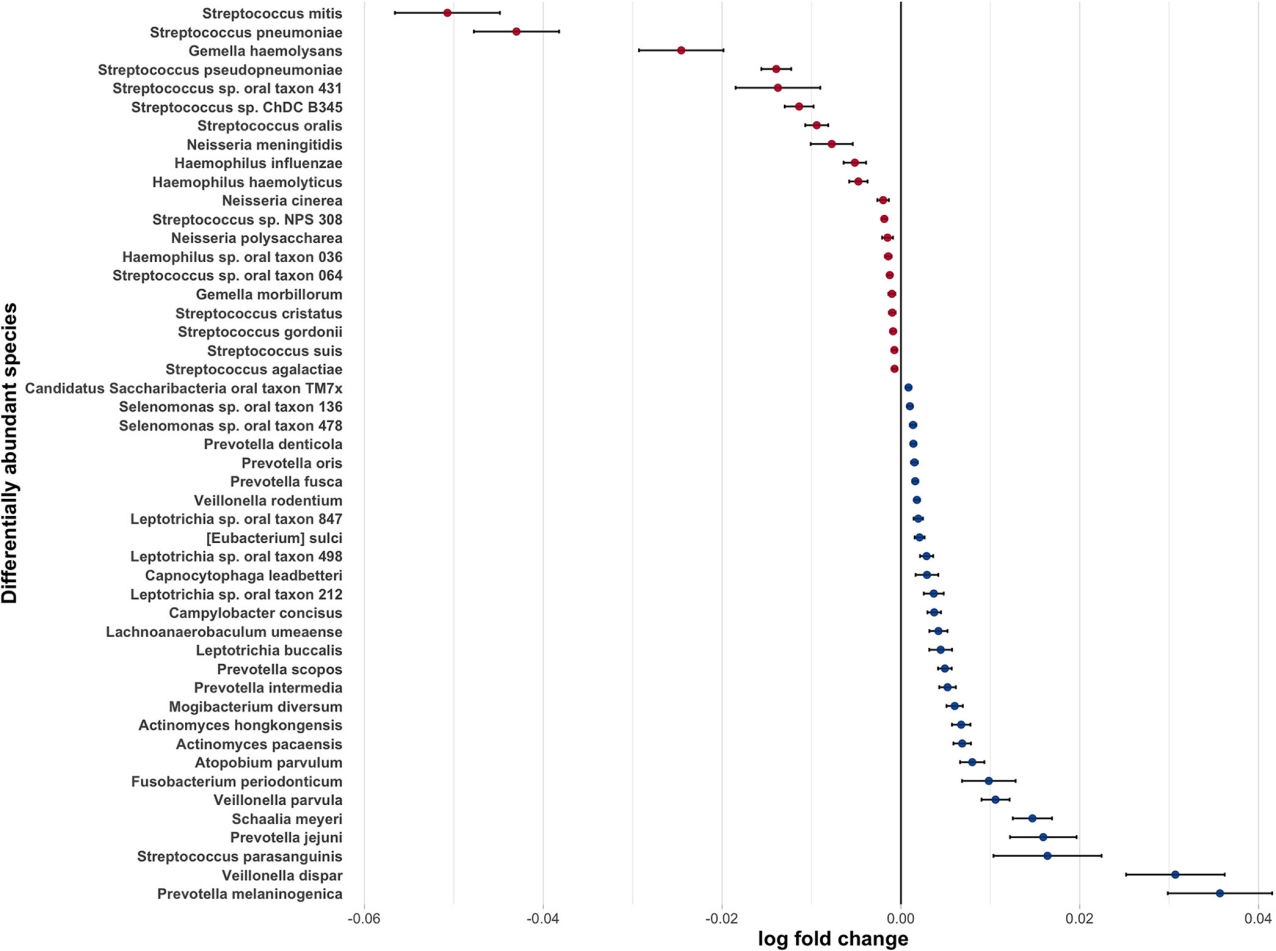
Species showing a significant difference in abundance in samples with a high versus low abundance of beta-lactam resistance genes. The relative abundances of species identified as differentially abundant, in samples with a high versus low abundance of beta-lactam resistance genes, by ANCOM-BC. Effect sizes were estimated via log fold (natural log) change in relative abundance of each species between high and low. Taxa more abundant in the high-beta-lactam RGPC group have an effect size shifted to the left and are shown in red, whereas taxa more abundant in the low-beta-lactam RGPC group have an effect size shifted to the right and are in blue.

We sought to gain insight into correlations between taxa and the abundance of beta-lactam ARGs. Figure 5 shows relationships between abundance of 92 microbial taxa, treatment strategy group, and quartiles of beta-lactam, macrolide, and multidrug RGPC. Individual OAV2 samples were classified into high- or low-abundance beta-lactam ARG groups based on whether the abundance of beta-lactam ARGs was above or below the median of all OAV2 samples. We then used ANCOM-BC to identify differentially abundant species in OAV2 samples with the high- versus low-beta-lactam ARGs. ANCOM-BC identified 48 differentially abundant taxa. Twenty taxa were more abundant in children with the higher levels of beta-lactam ARGs: 12 Streptococcus species (including Streptococcus pneumoniae), 3 Haemophilus species (including H. influenzae), 3 *Neisseria* species, and 2 *Gemella* species. In contrast, 7 *Prevotella* and 3 *Veillonella* species were identified among the 28 taxa that were more abundant in children with the lowest quartile of beta-lactam ARGs (Fig. 6).

### Abundance of gastrointestinal ARGs at the end of the study.

Stool samples were obtained and evaluated for ARGs at OAV2. Of the 171 participants who contributed throat samples, 74 also contributed stool samples at OAV2. The median age of participants who contributed stool and throat samples was younger than the median age of children who contributed only throat samples (25 months versus 43 months, *P* < 0.001) (Table S1). Racial groups also differed when comparing those who contributed stool and throat samples to those who contributed only throat samples. Ethnicity, initial antibiotic prescribed, and treatment strategy did not differ between the two groups. There were no significant differences identified for stool RGPC for any of the 10 ARG types when comparing the short course and standard course strategy groups (Fig. S2).

10.1128/mbio.00195-22.2 FIG S2 Boxplot of antibiotic resistance genes per prokaryotic cell (RGPC) for 10 clinically relevant antibiotic types in stool samples from 74 participants at the end of the study. The boxplots depict the distribution of RGPC for 10 clinically relevant antibiotic types. The line reflects the median RGPC, lower and upper hinges correspond to the first and third quartiles, and upper and lower whiskers extend from the hinge to the highest value that is within 1.5× interquartile range of the hinge. A one-sided Wilcoxon rank sum test was used to assess statistically significant differences (alpha level <0.05) between the numbers of RGPC in participants assigned to a short course (red) or standard course (blue) strategy. Log_2_ normalized RGPC data were used for visualization. Download FIG S2, DOCX file, 0.3 MB. Copyright © 2022 Pettigrew et al. 2022 Pettigrew et al. https://creativecommons.org/licenses/by/4.0/ This content is distributed under the terms of the Creative Commons Attribution 4.0 International license.

10.1128/mbio.00195-22.3 TABLE S1 Comparison of characteristics of the study participants who contributed stool and throat samples versus those who contributed throat samples only. Download Table S1, DOCX file, 0.01 MB. Copyright © 2022 Pettigrew et al. 2022 Pettigrew et al. https://creativecommons.org/licenses/by/4.0/ This content is distributed under the terms of the Creative Commons Attribution 4.0 International license.

## DISCUSSION

Defining the optimal duration of antibiotic therapy for pediatric CAP has been a priority area for child health research (9). Recent studies have shown that 5 days may be as effective as 10 days of therapy for uncomplicated CAP in children (6, 7). Our data show that the number of beta-lactam and multidrug efflux pump RGPC were significantly lower in children receiving short durations of beta-lactam antibiotics than in those who received standard durations of therapy for CAP at the end of the study (19 to 25 days after initiation of the study drug). The abundance of RGPC for 10 clinically relevant antibiotic types did not differ between treatment strategy groups at enrollment. Our temporal analyses of the resistome, which compared ARG abundances and the compositional profile of the resistome at enrollment and the end of the study, suggest that children receiving standard durations of beta-lactam antibiotics exhibit a greater abundance of ARGs for a longer period of time than do children receiving shorter durations of therapy. While we do not know how persistent these differences in the resistome are, these data suggest that reductions in the duration of exposure to beta-lactam therapy are associated with a lower abundance of ARGs in the respiratory microbiome. Thus, where possible and when clinical outcomes are similar, shorter durations of therapy are likely more desirable due to reduced antibiotic exposure and concomitantly lower levels of antibiotic selective pressure. Antibiotic selection pressure is linked to the prevalence of antibiotic resistance (14); thus, our results help inform the design of effective antibiotic treatment strategies that minimize selection of antibiotic resistance in bacteria.

In 2014 to 2015, the average annual number of antibiotic prescriptions for pneumonia was 931,748 (95% confidence interval, 627,845 to 1,235,652) for individuals <20 years of age (2). Beta-lactams are listed in the World Health Organization (WHO) Access group of antibiotics due to their effectiveness against a wide variety of commonly encountered pathogens (15). Widespread adoption of a 5-day beta-lactam strategy for the treatment of pediatric CAP could lead to a reduction in antibiotic exposure of approximately 5 million antibiotic days in U.S. children. Infection or colonization with antibiotic-resistant bacteria affects the risk of colonization and/or infection in others; reductions in antibiotic resistance have the potential to benefit both individual children and members in the community at large (16). While we did not examine this in our study, observational data suggest that reductions in the number of antibiotic doses result in lower levels of resistance in the community. Dagan and colleagues examined seasonality of antibiotic-resistant Streptococcus pneumoniae causing otitis media in Jewish and Bedouin children in Israel (17). Amoxicillin was the most commonly prescribed antibiotic in both populations. A 36% decline in antibiotic prescribing resulted in significantly lower rates of penicillin, erythromycin, and multidrug resistance in S. pneumoniae in Jewish children. Further studies are needed as relationships between levels of antibiotic use and antibiotic resistance are complex and depend on the bacterial species, mechanism of resistance (e.g., by mutation or horizontal transfer of resistance genes), and fitness costs of resistance.

Children in SCOUT-CAP were randomized to a difference in the duration of beta-lactam therapy and were evaluated at OAV2, which occurred approximately 19 to 25 days after the most recent antibiotic dose in the short course strategy group but 14 to 20 days after the most recent antibiotic dose in the standard course strategy group (Fig. 1). Thus, the observed differences in RGPC may be related to differences in the duration of treatment and/or temporal proximity of treatment to the sampling time. Regardless of the mechanism, even temporary reductions in the duration of microbiome dysbiosis are likely beneficial. Antibiotic-associated disruptions of the microbiota can increase susceptibility to colonization by new strains/resistant organisms (16). Higher densities of resistant organisms may lead to higher levels of shedding and transmission of resistant organisms (18, 19).

The children in our study were treated with beta-lactams, and we identified increases in resistance determinants in two different antibiotic classes (beta-lactams and multidrug efflux pumps). This observation suggests that some of the bacteria that were affected by beta-lactam therapy may have contained multiple resistance genes, which can result in cross-resistance. In theory, selection pressure with beta-lactams could result in an increase in the diversity of ARGs; however, we did not observe a significant difference in Shannon and inverse Simpson diversity at OAV2. Our observations suggest that the range of different types of antibiotic resistance genes in the resistome did not differ by group but that the abundance of individual ARG types did. We did not observe differences in the abundance of individual ARGs (i.e., ARG subtypes). However, the high dimensionality (i.e., large number of individual resistance genes identified compared to the number of children sampled) as well as the substantial sparsity (i.e., multiple zeroes in the subtype ARG data set due to absence of individual resistance genes in most samples) creates statistical challenges for identifying differentially abundant ARGs at the subtype level.

Hoberman et al. compared 5 days to 10 days of amoxicillin-clavulanate for the treatment of acute otitis media in children <2 years of age (20). Nasopharyngeal colonization was assessed by culture; there were no significant differences in the prevalence of nasopharyngeal colonization due to penicillin-nonsusceptible H. influenzae or S. pneumoniae between the two treatment groups. Very few randomized controlled trials have examined antibiotic-associated changes in the respiratory resistome in children (21). Keenan and colleagues evaluated the impact of macrolides on nasopharyngeal macrolide resistance using data from a cluster randomized trial of 24 communities in Niger undergoing annual or biannual mass treatment with azithromycin for trachoma (21). Communities with more frequent treatment had a higher prevalence of genetic macrolide resistance. This study examined *erm*B and *mef*A/E resistance determinants, and the impact of macrolides on other resistance genes is unknown.

LEfSe analyses of the microbiota indicated that seven different commensal species were differentially abundant when comparing the short course and standard course strategy groups. ANCOM-BC is conservative (22) and also identified *N. subflava* as more abundant in the standard course strategy group; however, this result was no longer significant after FDR correction. *N. subflava* is a commensal *Neisseria* species that has been shown to increase in abundance after experimental human respiratory tract infection (23). Commensal *Neisseria* also serves as a reservoir of resistance determinants and virulence genes for pathogenic *Neisseria* species (24, 25).

A few randomized controlled trials have examined the impact of antibiotics on the gut resistome (26, 27). We did not identify significant differences in the gastrointestinal resistome when comparing children randomized to short durations of beta-lactam antibiotics with those who received standard durations of therapy. Oldenburg et al. conducted a randomized trial of azithromycin, amoxicillin, co-trimoxazole, or placebo in children in Burkina Faso ages 6 to 59 months (26). Our results are consistent with their finding that beta-lactam resistance did not significantly differ by treatment group. However, our gastrointestinal resistome data should be interpreted with caution because the analysis was underpowered and the participants who contributed stool samples did not reflect the population recruited for the SCOUT-CAP trial.

Our study had limitations. The SCOUT-CAP study was limited to otherwise healthy children <6 years with outpatient CAP. Thus, the conclusions of our study may not extend to children with underlying conditions or other populations of children with high levels or prior antibiotic use. The resistome was evaluated at the end of the study, and the duration of follow-up was short (approximately 1 month after the initiation of antibiotic treatment), and the observed changes in the resistome may be transient. The impact of antibiotics on the microbiome differs by the type of antibiotic and the body site (21, 28, 29). Children in SCOUT-CAP were prescribed one of three beta-lactams as their initial antibiotic. In *post hoc* analyses, we compared the abundances of ARG types by treatment strategies at the end of the study restricted to the 156 children initially prescribed amoxicillin. Results were consistent with the ITT analysis; beta-lactam, macrolide, and multidrug efflux RGPC were lower in children receiving the short compared to standard treatment strategy (Wilcoxon rank sum test, *P* = 0.03, 0.04, and 0.004, respectively). However, only multidrug efflux RGPC were significantly lower after FDR adjustment for multiple comparisons (*P* = 0.04). The small number of children prescribed amoxicillin-clavulanate (*n* = 11) and cefdinir (*n* = 4) precluded meaningful comparisons by treatment strategy in these subgroups. The design of our study allowed identification of genes associated with resistance, and some genes may not be expressed.

A major strength of these data is that they are from a multicenter, randomized, double-blind, placebo-controlled, superiority clinical trial. To our knowledge, there have been no studies to date that have evaluated relationships between duration of antibiotic use and the respiratory resistome. We demonstrated that children receiving 5 days of beta-lactam therapy for CAP had a significantly lower prevalence of two different types of ARGs than did those receiving the standard 10-day treatment. These data provide an additional rationale for reductions in antibiotic use that can disrupt microbial communities and lead to adverse events following antimicrobial treatment. Trials are increasingly examining the efficacy of shorter durations of antibiotic treatment for a range of diseases (20, 30–32). Future studies of the efficacy and duration of antibacterials should consider the impact on the microbiota and resistome as part of the trial design.

## MATERIALS AND METHODS

### Study population.

SCOUT-CAP study subjects were children 6 to 71 months of age diagnosed with CAP and prescribed either amoxicillin, amoxicillin-clavulanate, or cefdinir by a health care provider in an ambulatory care setting at one of eight study sites (7). Eligible subjects were screened and enrolled between 2 December 2016 and 16 December 2019. Subjects were included in the current study if they consented and enrolled in SCOUT-CAP and also consented to the collection of throat swabs and/or stool samples for future use. The future-use microbiome study protocol was reviewed and approved by the Yale University and Duke University institutional review boards.

### Data and samples.

The study procedures for SCOUT-CAP have been described previously (7). Briefly, SCOUT-CAP participants were assessed for eligibility and enrolled on day 3 to 5 of the oral beta-lactam therapy (Fig. 1). Participants were randomized 1:1 to receive either 5 days of placebo (short course) or 5 additional days of their initially prescribed antibiotic (standard course). Data regarding baseline demographics, medical history, and concomitant medications were collected at enrollment. Caregivers were also provided a memory aid to keep a daily record of the antibiotics and other medications that were taken during the study. Samples included in the current study included throat swabs taken by study personnel and stool samples submitted at (i) the enrollment visit, which occurred 3 to 5 days after starting antibiotics for CAP, and (ii) the outcome assessment visit (OAV2), which occurred at the end of study on days 19 to 25 after initiation of the study drug.

### DNA extraction and shotgun metagenomics sequencing.

DNA was extracted from throat and stool specimens using the PureLink microbiome DNA purification kit (Invitrogen, Carlsbad, CA). Shotgun sequencing libraries were prepared using the NEBNext Ultra II FS DNA library prep kit with sample purification beads for Illumina using the protocol for inputs of ≤100 ng. The adaptor-ligated DNA was amplified for eight PCR cycles using NEBNext multiplex oligonucleotides for Illumina. The individual libraries were pooled in equal nanogram amounts and sequenced using 150-bp paired-end sequencing at the Yale Center of Genome Analysis on the Illumina HiSeq 4000 or NovaSeq.

### Characterization of the resistome and microbiota.

We used Btrim software to sort, trim, and filter out low-quality sequence reads (33). The average number of total paired reads per throat sample and standard deviation (SD) were 21,263,314 ± 8,482,865. After trimming, the mean number and SD of reads were 20,598,786 ± 8,363,319. The ARG online analysis pipeline (OAP) v2.0 with the expanded structured antibiotic resistance genes (SARG) database was used for classification and quantification of ARGs (34). The pipeline contains reference sequences that encode over 1,200 distinct resistance subtypes and provides quantification of reads for individual ARGs (referred to as ARG subtypes). ARGs are also grouped into categories, referred to as ARG types, which involve categorization as “unclassified” resistance genes or by resistance to each of 23 different ARG types (e.g., beta-lactam resistance) (34). Data were normalized against prokaryotic cell numbers, and ARG abundances were provided at the type and subtype level as resistance genes per prokaryotic cell (RGPC) (34).

The Kraken 2 pipeline, a k-mer-based approach, was used for taxonomic identification of shotgun metagenomic sequence reads (35). Kraken 2-based taxonomic assignments were then used in Bracken (Bayesian Reestimation of Abundance after Classification with KrakEN) to estimate the species-level abundance of taxa within each sample (36).

### Statistical analyses.

Statistical analyses were conducted using R 4.0.2 (R Foundation for Statistical Computing), and plots for visualization were created using ggplot2 (37). Baseline and demographic characteristics were summarized overall and by treatment group. Unadjusted associations between individual patient characteristics and treatment strategy were compared by chi-square test, Fisher’s exact test, Wilcoxon rank sum, and Wilcoxon signed-rank test as appropriate. Wilcoxon effect sizes (*r*) and 95% confidence intervals (CIs) were calculated using the “wilcox_effsize” function in rstatix and implemented in R (38). Wilcoxon effect sizes were interpreted using criteria similar to those used for Cohen’s *d* and were as follows: <0.05 = very small, 0.1 = small, 0.2 = medium, 0.3 = large, and 0.4 = very large (39).

The primary prespecified endpoint for the resistome analysis was the number of beta-lactam RGPC in throat swabs collected at OAV2. The null hypothesis was that the distribution of beta-lactam RGPC in the two study strategies would be the same. The alternative hypothesis was that children randomized to the short strategy would have fewer type-level beta-lactam resistance genes detected. A one-sided Wilcoxon rank sum test was conducted to assess whether there were statistically significant differences (alpha level <0.05) in the number of RGPC in children in the short versus standard strategy groups. Sample size estimates were based on preliminary data on the distribution of beta-lactamase resistance genes from 57 throat samples that were blinded to treatment group assignment. Based on the distribution of beta-lactamase gene data in the 57 throat samples, a sample size of 200 total subjects (100 in each group) would achieve 75% power to detect a shift of 12% of data from the lower half of the distribution to the upper half of the distribution between the two treatment strategies using a one-sided α = 0.05. The primary analysis was carried out with an ITT principle, and analyses were done per randomized treatment assignment.

Additional exploratory analyses comparing the abundances of RGPC between treatment strategies focused on a subset of nine more of the 24 ARG types, selected for clinical relevance: aminoglycoside, macrolide-lincosamide-streptogramin (here referred to as macrolide), multidrug efflux pumps, quinolone, rifamycin, sulfonamide, tetracycline, trimethoprim, and vancomycin. The selection of the nine additional ARG types was done prior to the commencement of data analysis. Analyses of the nine additional ARG types were as described above for beta-lactam resistance genes, and the Benjamini-Hochberg procedure was used to derive FDR-adjusted *P* values to correct for multiple comparisons (40).

Diversity indices are mathematical measures of the number of different types of species and also the relative abundance of species in a given community. Two alpha diversity indices, Shannon-e (natural log) (41) and Simpson’s reciprocal index (42), were calculated using all ARG type data or all species-level Bracken data for each sample using phyloseq (43) and vegan (44) R packages. Bray-Curtis dissimilarity is a beta diversity measure used to quantify the compositional dissimilarity between samples (45). We calculated Bray-Curtis dissimilarity to measure the compositional dissimilarity in abundance and presence of all type-level ARGs at enrollment and OAV2. The dissimilarity indices were used to create ordination plots, and PCoA was used to graphically depict differences in ARG community profiles by visit and treatment strategy (46). PERMANOVA (47) was used to evaluate the statistical significance of the differences in the composition of ARGs across treatment strategies iterated over 10,000 permutations; we handled multiple comparisons by using the Benjamini-Hochberg procedure to derive FDR-adjusted *P* values (40). We used two different differential abundance analysis methods to identify potentially discriminant taxa: LEfSe (12) and ANCOM-BC (13). We applied uniform filtering to remove all-zero taxa and selected species with a minimum prevalence of 10% and a minimum abundance of 10^−3^ prior to differential abundance analyses.

### Data availability.

The data that support the findings of this study are available from the corresponding author upon request; limitations apply to variables that may compromise participant privacy or consent. The shotgun metagenomic sequencing reads have been deposited with the NCBI Sequence Read Archive and are available under BioProject PRJNA745160.
