# Supplementary material for: Comparison of the Respiratory Resistomes and Microbiota in Children Receiving Short versus Standard Course Treatment for Community-Acquired Pneumonia
Source: mBio. 2022 Mar 24;13(2):e00195-22. doi: 10.1128/mbio.00195-22 (PMC9040816; doi:10.1128/mbio.00195-22)
Supplement: FIG S1 [file mbio.00195-22-sf001.docx]

Supplemental Figure 1. Boxplot of respiratory resistance genes per prokaryotic cell (RGPC) for 10 clinically relevant ARG types in throat swabs at enrollment (N=158)

**
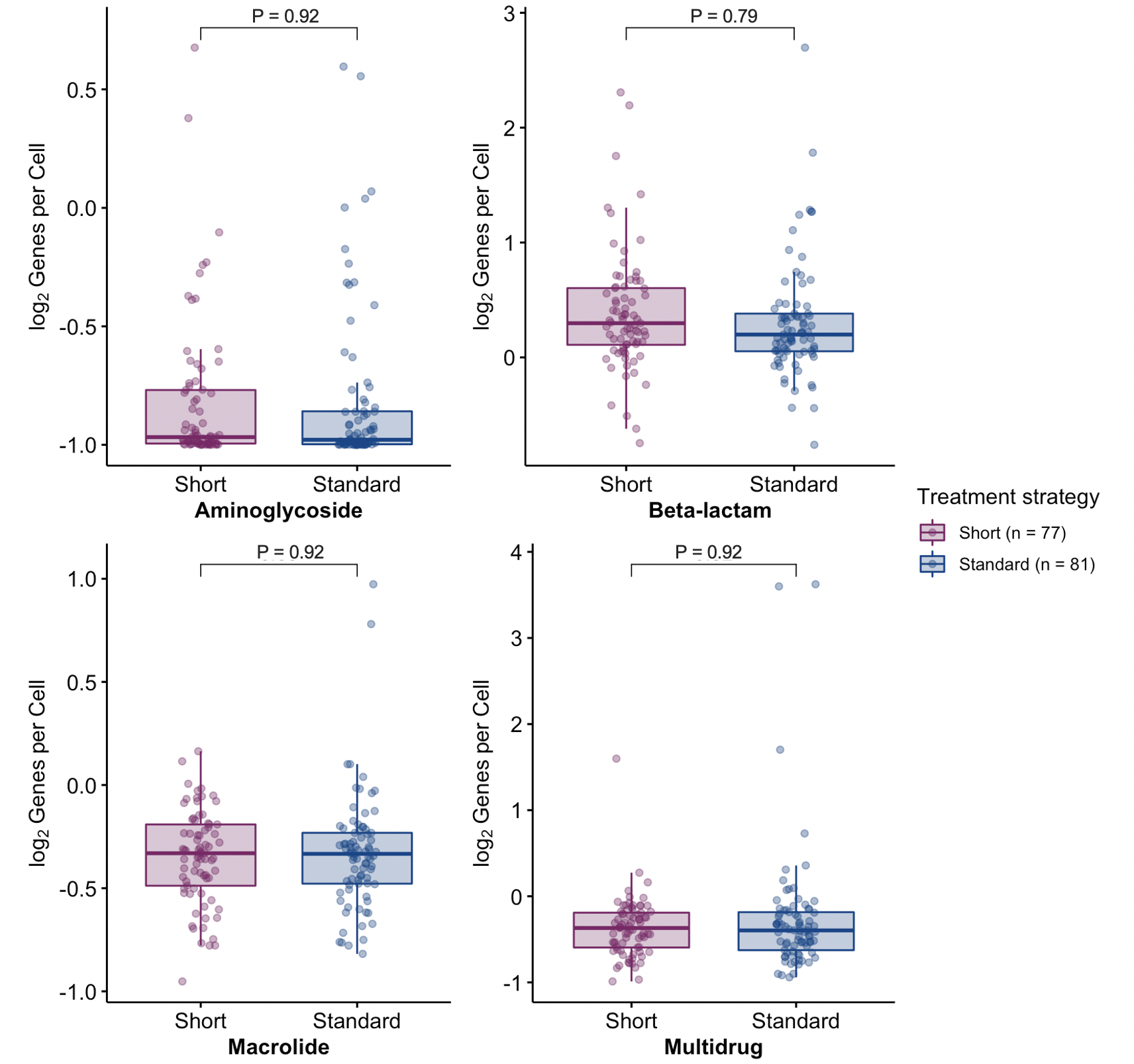
**

**
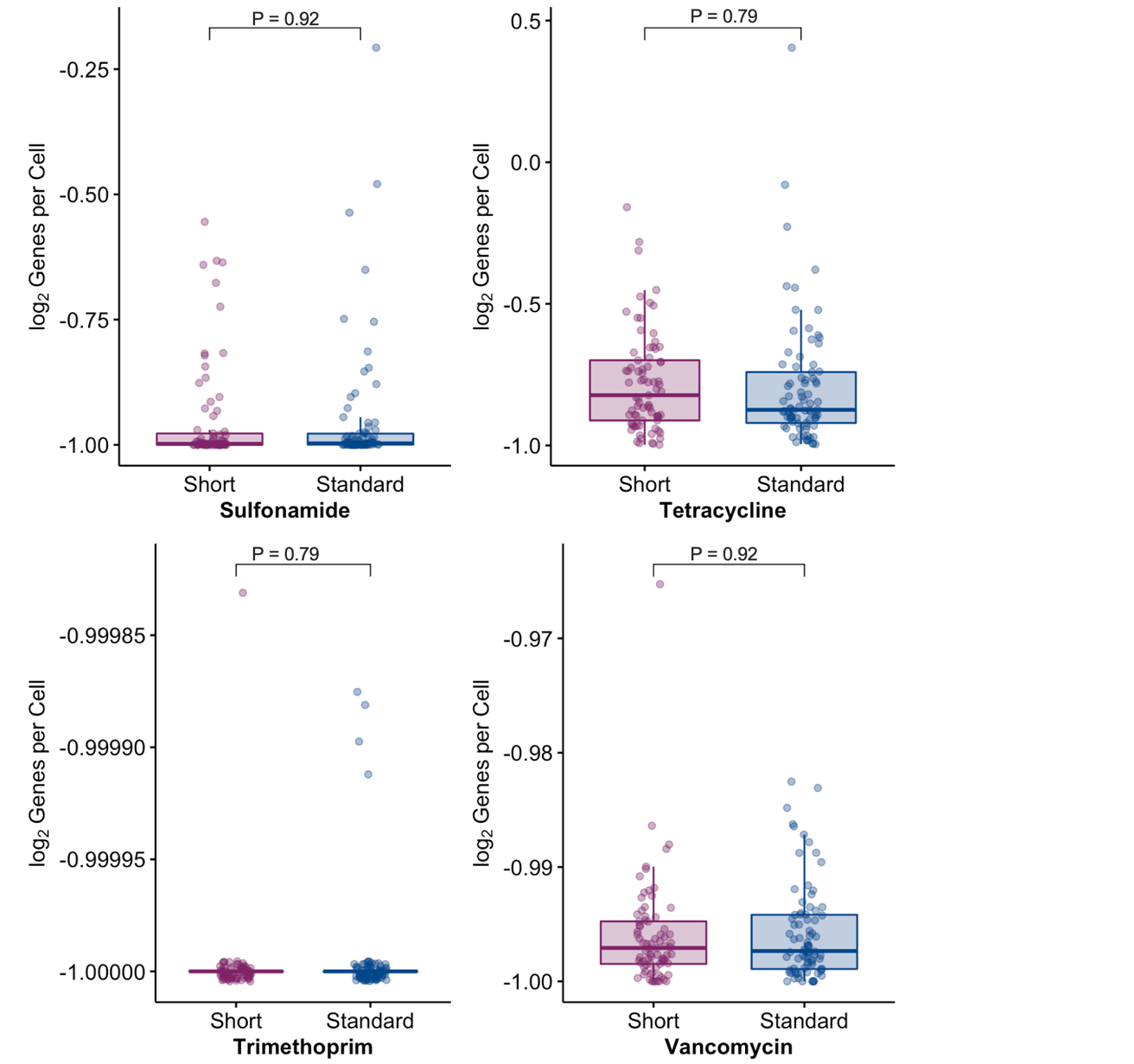
**
