# Supplementary material for: Comparison of the Respiratory Resistomes and Microbiota in Children Receiving Short versus Standard Course Treatment for Community-Acquired Pneumonia
Source: mBio. 2022 Mar 24;13(2):e00195-22. doi: 10.1128/mbio.00195-22 (PMC9040816; doi:10.1128/mbio.00195-22)
Supplement: FIG S2 [file mbio.00195-22-sf002.docx]

Supplemental Figure 2. Boxplot of antibiotic resistance genes per prokaryotic cell (RGPC) for 10 clinically relevant antibiotic types in stool samples from 74 participants at the end of the study.

**
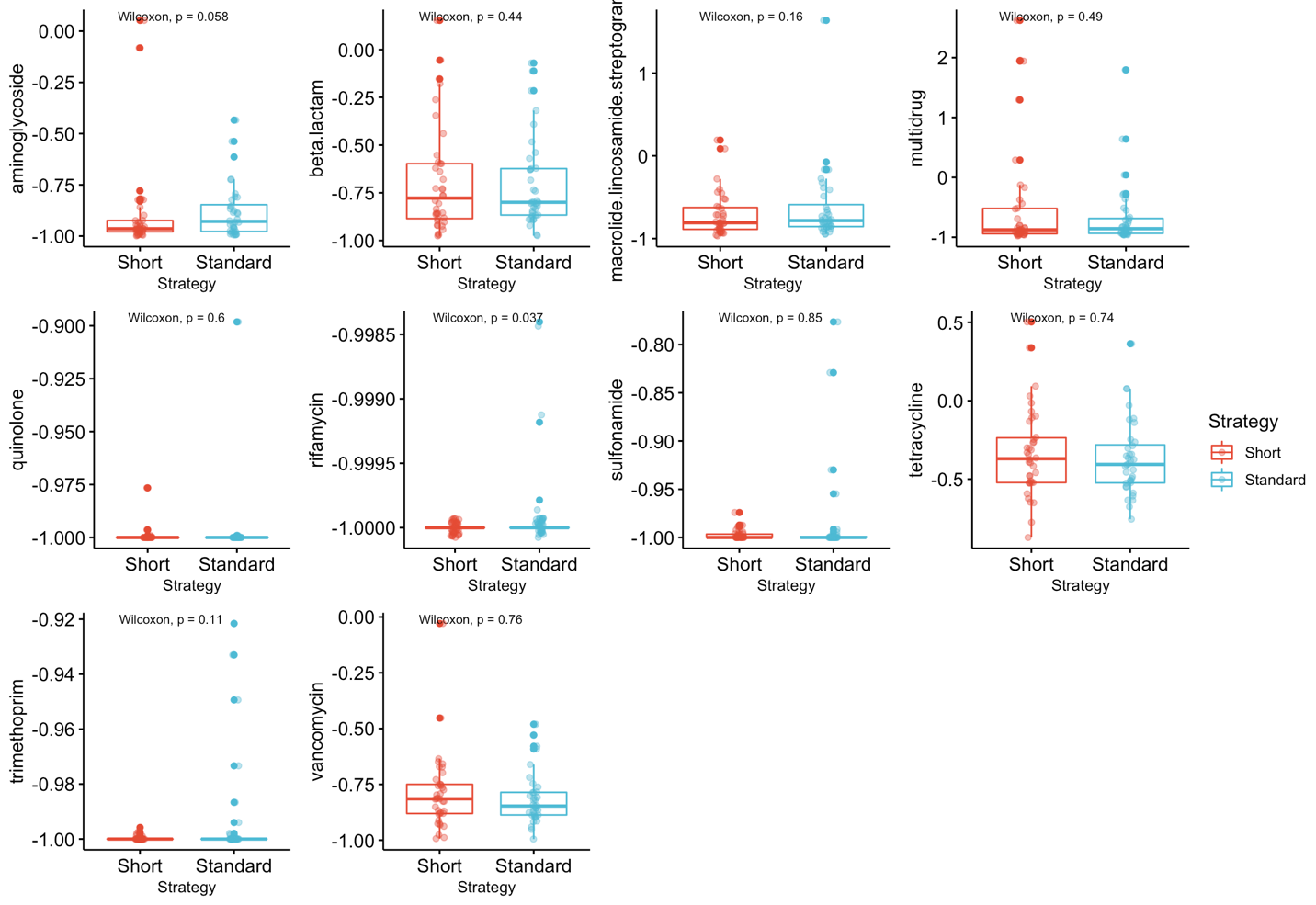
**
