# Supplementary material for: Comparison of the Respiratory Resistomes and Microbiota in Children Receiving Short versus Standard Course Treatment for Community-Acquired Pneumonia
Source: mBio. 2022 Mar 24;13(2):e00195-22. doi: 10.1128/mbio.00195-22 (PMC9040816; doi:10.1128/mbio.00195-22)
Supplement: TABLE S1 [file mbio.00195-22-st001.docx]

Supplemental Table 1. Comparison of characteristics of the study participants who contributed stool and throat samples vs. those who contributed throat samples only.

| **Characteristic** | **Stool population (N=74)**  **n (%)** | **Throat population**  **(N = 97)**  **n (%)** | **P-value** |
| --- | --- | --- | --- |
| **Age** |  |  | **<0.001** |
| 6 to 23 months | 35 (47) | 21 (22) |  |
| 24-71 months | 39 (53) | 76 (78) |  |
| Median (months) | 25 | 43 |  |
| **Sex** |  |  | 0.84 |
| Female | 37 (50) | 47 (48) |  |
| Male | 37 (50) | 50 (52) |  |
| **Race** |  |  | **0.02** |
| Asian | 2 (3) | 3 (3) |  |
| Black or African American | 10 (14) | 32 (33) |  |
| Multi-racial | 6 (8) | 6 (6) |  |
| White | 56 (76) | 53 (55) |  |
| Unknown | 0 (0) | 3 (3) |  |
| **Ethnicity** |  |  | 0.42 |
| Hispanic or Latino | 9 (12) | 9 (9) |  |
| Not Hispanic or Latino | 64 (86) | 88 (91) |  |
| Unknown | 1 (1) | 0 (0) |  |
| **Initial antibiotic** |  |  | 0.86 |
| Amoxicillin | 68 (92) | 88 (91) |  |
| Amoxicillin-clavulanate | 4 (5) | 7 (7) |  |
| Cefdinir | 2 (3) | 2 (2) |  |
| **Treatment Strategy Group** |  |  | 0.61 |
| Short Course Strategy | 38 (51) | 46 (47) |  |
| Standard Course Strategy | 36 (49) | 51 (53) |  |
|  |  |  |  |
